# Privacy and utility of genetic testing in families with hereditary cancer syndromes living in three countries: the international cascade genetic screening experience

**DOI:** 10.3389/fgene.2023.1109431

**Published:** 2023-05-09

**Authors:** Sivia Barnoy, Efrat Dagan, Sue Kim, Maria Caiata-Zufferey, Maria C. Katapodi, the CASCADE, the K-CASCADE Consortia

**Affiliations:** ^1^ Department of Nursing, Tel-Aviv University, Tel-Aviv, Israel; ^2^ The Cheryl Spencer Department of Nursing, University of Haifa, Haifa, Israel; ^3^ College of Nursing, Yonsei University, Seoul, South Korea; ^4^ Department of Business Economics, Health and Social Care, University of Applied Sciences and Arts of Southern Switzerland, Manno, Switzerland; ^5^ Department of Clinical Research, University of Basel, Basel, Switzerland

**Keywords:** cascade genetic testing, genetic health disparities, HBOC, Lynch syndrome, national healthcare system, patient-mediated dissemination, provider-mediated dissemination, public health genetic screening

## Abstract

**Background:** Hereditary breast and ovarian cancer and Lynch syndrome are associated with increased lifetime risk for common cancers. Offering cascade genetic testing to cancer-free relatives of individuals with HBOC or LS is a public health intervention for cancer prevention. Yet, little is known about the utility and value of information gained from cascade testing. This paper discusses ELSI encountered during the implementation of cascade testing in three countries with national healthcare systems: Switzerland, Korea, and Israel.

**Methods:** A workshop presented at the 5th International ELSI Congress discussed implementation of cascade testing in the three countries based on exchange of data and experiences from the international CASCADE cohort.

**Results:** Analyses focused on models of accessing genetic services (clinic-based versus population-based screening), and models of initiating cascade testing (patient-mediated dissemination versus provider-mediated dissemination of testing results to relatives). The legal framework of each country, organization of the healthcare system, and socio-cultural norms determined the utility and value of genetic information gained from cascade testing.

**Conclusion:** The juxtaposition of individual versus public health interests generates significant ELSI controversies associated with cascade testing, which compromise access to genetic services and the utility and value of genetic information, despite national healthcare/universal coverage.

## Introduction

Approximately a decade ago the Centers for Disease Control and Prevention (CDC) categorized hereditary breast and ovarian cancer (HBOC) and Lynch syndrome (LS) as Tier-1 genetic conditions based on the validity of genetic testing results and the utility of genetic information for healthcare practitioners and public health (Khoury and Evans, 2015). HBOC accounts for 5%–10% of all breast cancer and 25% of all ovarian cancer cases (Couch et al., 2014; Jonsson et al., 2019; Yoshida, 2021). Women with HBOC-associated variants have 45%–66% cumulative lifetime risk for breast cancer and 11%–41% cumulative risk for ovarian cancer by age 70, while the corresponding lifetime risks in the general population are 12% and 1.3%, respectively (Mavaddat et al., 2013; Couch et al., 2014; SEER., 2017). HBOC is also associated with increased lifetime risk for pancreatic cancer, and increased risk for prostate and male breast cancer (Kuchenbaecker et al., 2017; Jonsson et al., 2019). Similarly, individuals with LS-associated variants have a 10%–74% cumulative lifetime risk for colorectal cancer and a 14%–71% cumulative lifetime risk for endometrial cancer by age 70, while the corresponding rates in the general population are 5.5% and 2.7%, respectively (Weiss et al., 2021).

HBOC and LS are monogenic disorders caused by pathogenic variants in autosomal dominant genes (Weiss et al., 2021). HBOC- and LS-associated variants can be identified through panel testing. Targeted testing is almost 100% accurate in identifying the familial pathogenic variants among at-risk relatives, and costs significantly less (Kraus et al., 2017; Salikhanov et al., 2022). CDC recommended cascade testing in asymptomatic biological relatives of individuals with HBOC- or LS-associated variants as a public health intervention that can prevent cancer and reduce lifelong risk of adverse health outcomes (Khoury and Dotson, 2021). Various evidence-based strategies, such as annual breast MRI and colonoscopy, chemoprevention, and risk-reducing salpingo-oophorectomy, can reduce HBOC- and LS-associated morbidity and mortality (Paluch-Shimon and Cardoso, 2016; Crosbie et al., 2019; Forbes et al., 2019; Stjepanovic et al., 2019).

Cascade testing can reduce disease risks to entire cohorts of at-risk relatives (Umans-Eckenhausen et al., 2001; Bokkers et al., 2022), but also creates an increasing demand for genetic counseling and testing services, surveillance and follow-up care, and risk reduction interventions (Grosse et al., 2009; Nikolaidis et al., 2018). Genetic services are not equally accessible to all, with significant disparities observed based on sex, age, race and ethnic minority status, and place of residence (Manrriquez et al., 2017; Willis et al., 2017; Stamp et al., 2019). Advances in genomic technologies do not reach all segments of the population (National Academies of Sciences E and, 2018). Barriers relate to the organization of healthcare systems and finance structures, societal and cultural aspects, and individual factors, raising significant ethical and legal concerns about equity in accessing services (Dwyer et al., 2022).

In order to fully understand the value of genetic and genomic interventions for the individual, family, and society, to upscale the implementation of cascade testing programs, and to ensure a global benefit from technological advances in genomics, a meaningful consideration of ELSI associated with such interventions is required. This paper focuses on ELSI identified in Switzerland, Korea, and Israel from the implementation of cascade testing for HBOC and LS. The three countries have national healthcare systems (or national insurance plans) that entitle all citizens to a basket of services. National laws for the protection of genetic information are similar to the US Genetic Information Non-Discrimination Act (GINA) (Israel-genetic Information Law, 2001; Swiss Confederation-Federal Act on Human Genetic Testing, 2004; Republic of Korea-Bioethics and Safety Act of 2005, 2019). The specific aims of the paper are to discuss access to cancer genetic services and dissemination of genetic information to at-risk relatives for initiating cascade testing, and how they influence the utility of genetic information in the three countries.

## Methods

The international CASCADE cohort was initiated in Switzerland in 2016, and was implemented in Korea and Israel in 2020 and 2022, respectively. CASCADE is a family-based, open-ended cohort focusing on HBOC (and LS for Switzerland and Israel). The cohort has been approved by local ethics committees (BASEC 2016-02052, YUHS 4-2020-0520, RBM-0184-21, TLV-0162-21) and the protocol has been published (NCT03124212; NCT04214210) (Katapodi et al., 2017). Settings include university and cantonal hospitals and private practices that are geographically dispersed within each country, serve different linguistic regions, and cover approximately 50% of the population. Recruitment is initiated through index cases (first person in the family identified with an HBOC- or LS-associated variant). Index cases are asked to recruit as many relatives they are willing to contact. Relatives can be first-, second-, or third-degree; affected by cancer or cancer-free; and can be untested, can carry the familial pathogenic variant, or can be true negatives.

Rates of cascade testing are assessed with self-administered questionnaires that are available in local languages. Additional outcomes include cancer status and surveillance, risk-reducing surgeries, coordination of medical care, psychosocial and family factors, and quality of life. Participants are asked to complete a questionnaire after they provide written consent and they enter the cohort (baseline questionnaire). They are asked to complete follow-up questionnaires on a continuous basis, approximately 24 months apart, for as long as the study is running or for as long as they want to remain in the cohort. In Switzerland and in Korea narrative data are also collected from a purposeful sub-sample of participants, to get an in-depth understanding about dissemination of genetic information and access to services (Pedrazzani et al., 2022).

On 31st May 2022 a workshop presented at the 5th International ELSI Congress discussed insights from the international CASCADE cohort based on discussions among the study investigators and initial responses from index cases and relatives. The cohort was initiated in Switzerland, which had acquired most data at the time of the workshop. The development of the Swiss CASCADE and rates of genetic testing among relatives have been presented (Sarki et al., 2022a; Sarki et al., 2022b). In short, the Swiss CASCADE had consented 365 index cases (276 HBOC and 89 LS) and 158 relatives (140 HBOC and 18 LS). At the time of the workshop, data were available from 287 index cases and 115 relatives. K-CASCADE had recruited index cases and relatives, but at the time of the workshop data were available only for *n* = 91 tested individuals. Israel was in the first stages of recruitment and data from the Israeli cohort were not available.

The workshop focused on presenting ELSI associated with models of accessing genetic services and disseminating genetic information to at-risk relatives that are in effect in Switzerland, Korea, and Israel. Comparison of contextual factors is an important first step in understanding rates of cascade testing and management of hereditary cancer risk in the three countries. This paper provides a synopsis of the discussion addressing each specific aim, synthesized into concrete key points and supported by references. Hence, only data relevant to ELSI are presented.

## Results

### Access to cancer genetic services in national healthcare systems

The most common model for offering genetic services is the “clinic-based” model. Genetic specialists determine eligibility for services based on personal and/or family history indicating a hereditary cancer syndrome. Many countries require genetic counseling prior to and after genetic testing by law. Providers are the main “gate-keepers” and access to genetic services depends on referrals. Self-referrals are also possible, with cost-coverage depending on individual insurance plans. Costs of testing range from $300-$3,600 for targeted testing and full sequencing, respectively. Co-payments vary from 10% to 60%; in Israel co-payments can be 0% for individuals of Ashkenazi Jewish background. Co-payments of testing relatives range from 0% to 100% depending on family history and degree of relationship with the index case. Costs for risk-reducing surgeries for individuals with pathogenic variants are also not equally covered, i.e., risk-reducing mastectomy for unaffected carriers is not covered in Korea.

**FIGURE 1:**
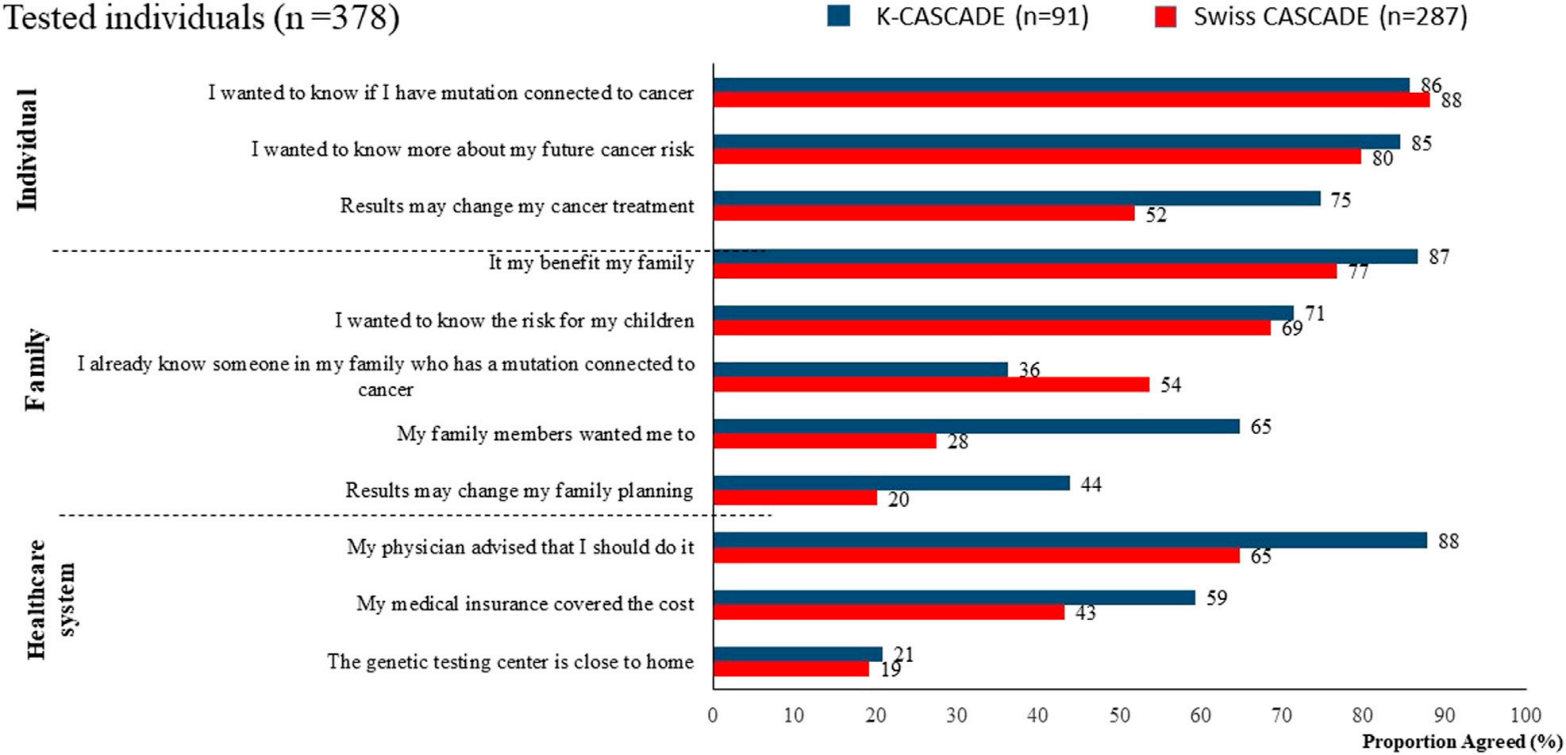
Reasons for having genetic testing among Swiss and Korean participants.

Variability in coverage of testing and risk-reducing strategies may contribute to decisional conflict in relatives about cascade testing, and to disparities in preventing and managing hereditary cancer, even in national healthcare systems. Data from *n* = 378 tested individuals from the CASCADE cohort (287 from Switzerland and 91 from Korea) show that more than 65% in both countries reported having genetic testing because it was recommended by a healthcare provider. Other common reasons for having genetic testing related to knowing about one’s personal cancer risk and family benefits (Figure 1).

**FIGURE 2:**
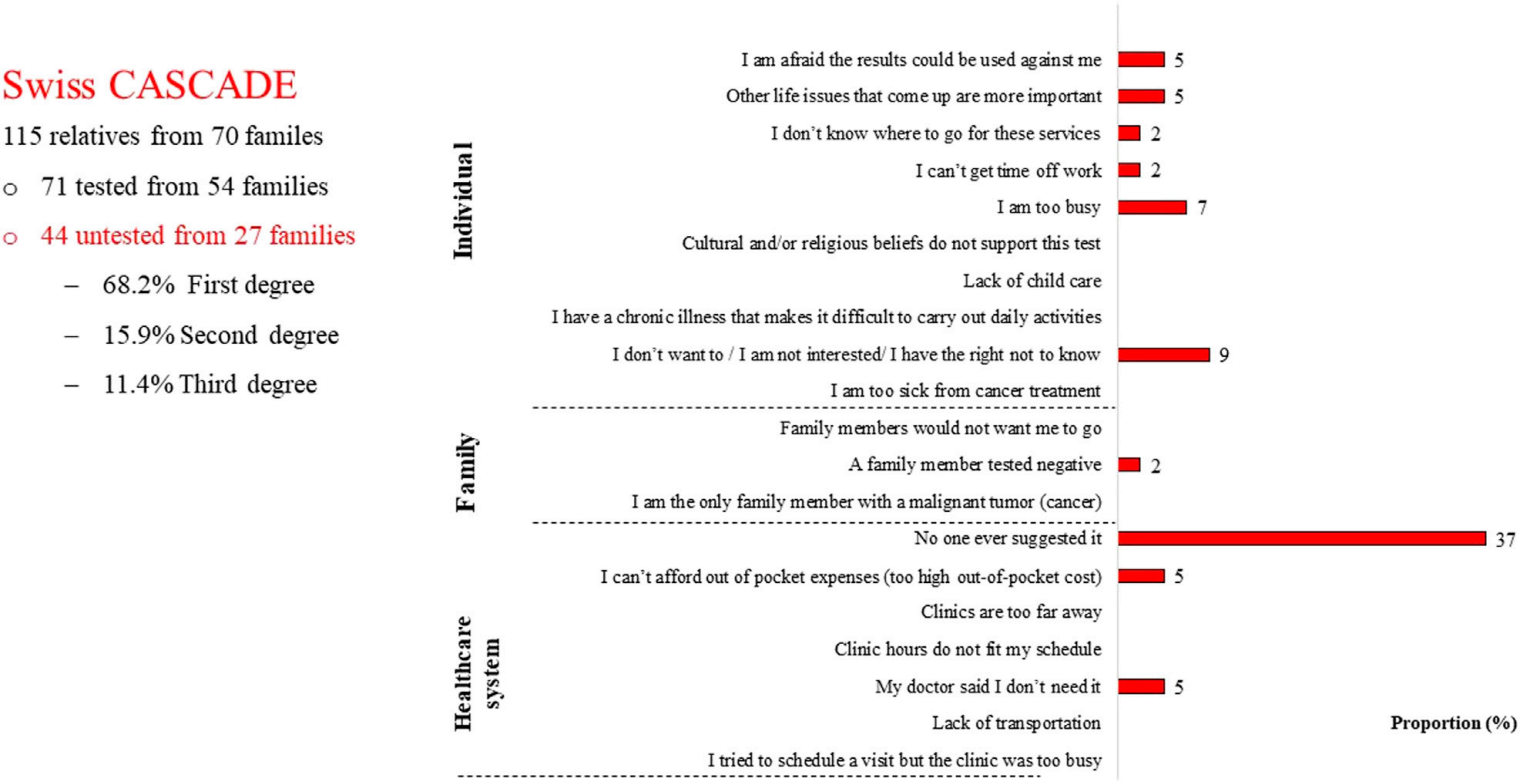
Reasons for not having genetic testing among Swiss relatives.

Among 115 relatives who had provided data to the Swiss CASCADE by December 2021, 38.3% (*n* = 44) did not have cascade testing; among untested relatives 68.2% (*n* = 30) were first-degree relatives and 93% (*n* = 41) were cancer free (Sarki et al., 2022b). Surprisingly, these *n* = 44 untested relatives reported that the main reason for not having testing was lack of a provider referral (Figure 2).

**FIGURE 3:**
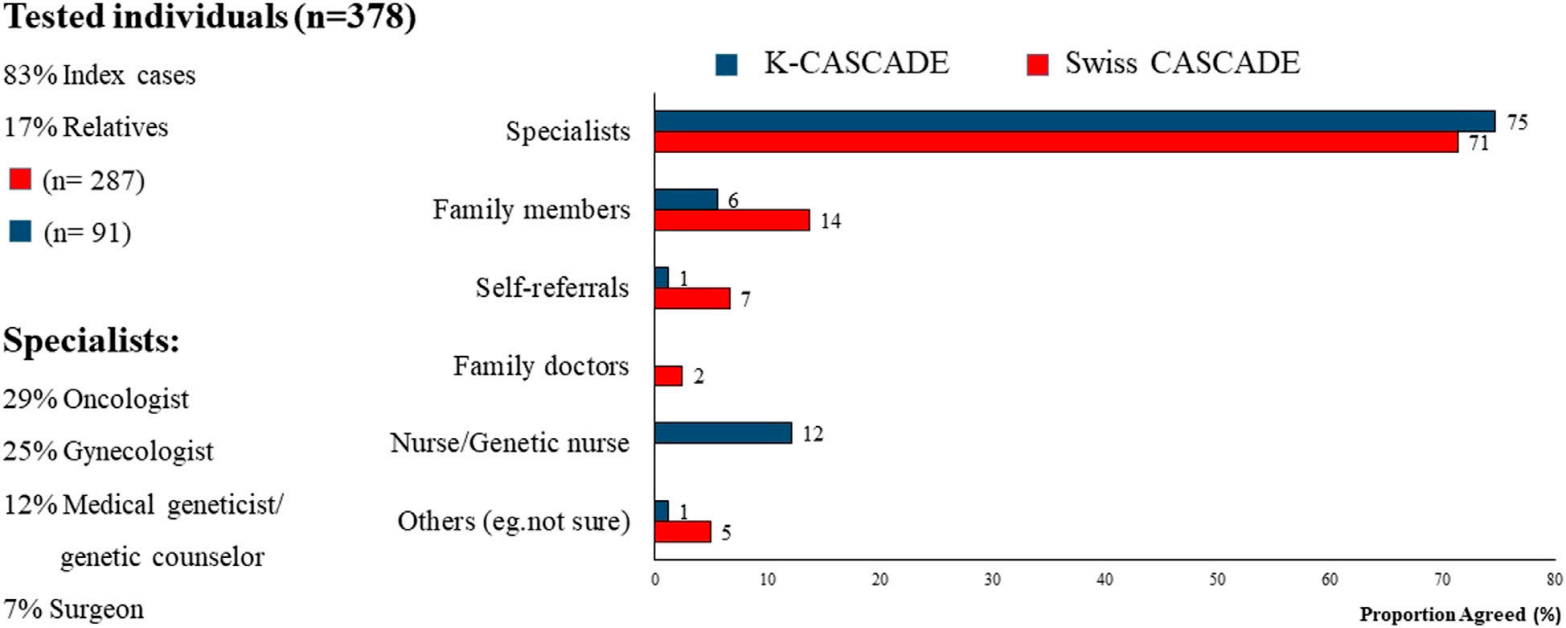
Sources of referral for genetic testing for Swiss and Korean participants.

Approximately 70% of index cases in Switzerland and Korea reported that referrals to genetic services were provided primarily by specialists. Among them, 29% stated that the specialist was an oncologist, suggesting that the referral was provided after a cancer diagnosis. There was minimal contribution to referrals from family doctors and general practitioners, suggesting lack of collaboration between primary care and genetic services with possible delays in identifying and evaluating cases at-risk for HBOC or LS (Figure 3). Self-referrals were fewer compared to the US (up to 7% compared to 12%) (Stamp et al., 2019).

**FIGURE 4:**
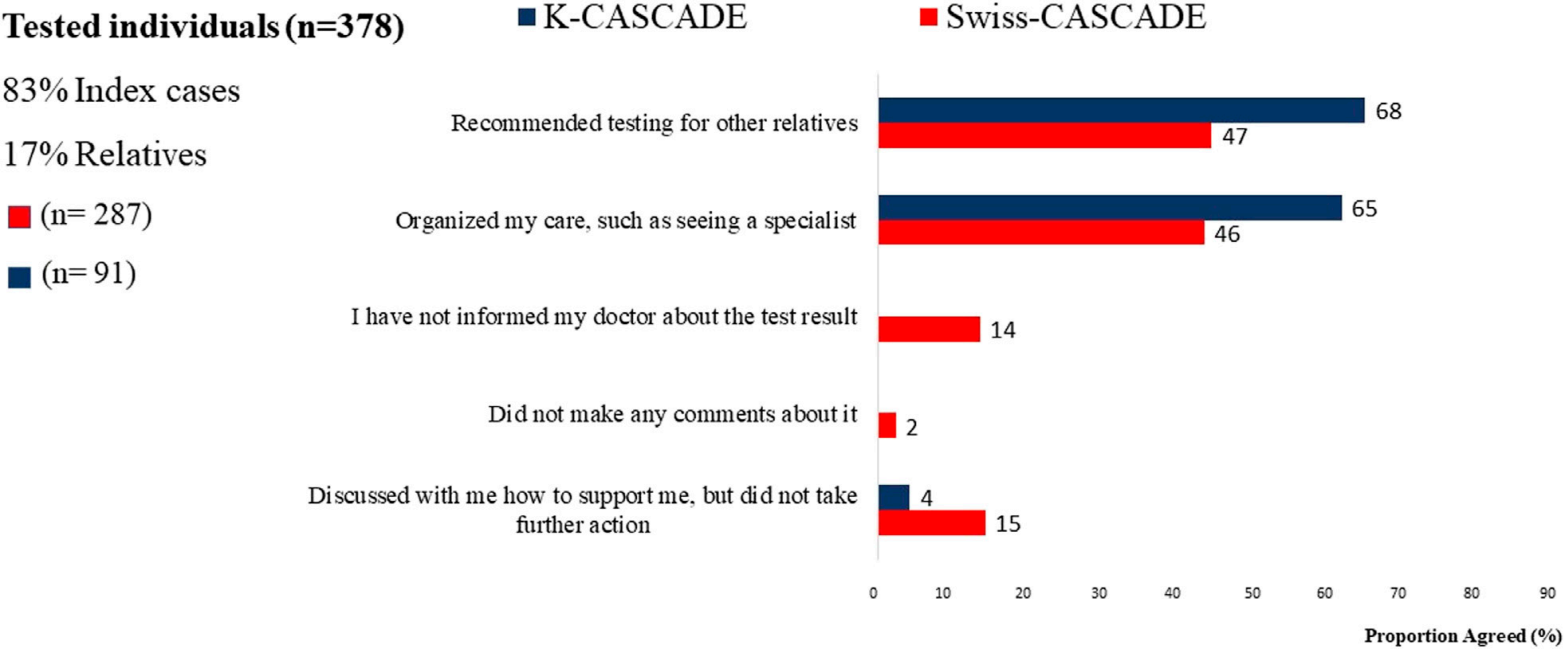
Respond of Clinician after genetic testing reported by Swiss and Korean participants.

Many individuals who had genetic testing in Switzerland and Korea reported receiving recommendations for cascade testing in relatives. However, 53% (*n* = 152) of Swiss and 32% (*n* = 29) of Korean tested individuals did not remember receiving such a recommendation (Figure 4). This finding is based on self-report and the study cannot verify whether tested individuals received or did not receive recommendations for cascade testing in relatives. Nevertheless, it indicates significant missed opportunities for encouraging dissemination of genetic information to untested relatives in both countries.

Israel implements a “population-based” genetic screening program for identifying individuals with HBOC-associated variants because approximately 30% of the population is of Ashkenazi Jewish background and potentially one in 40 carry such a variant. The program targets specifically women, who can be self-referred to genetic services, while the cost of testing is covered (0% co-payment) (Michaan et al., 2021). The program capitalizes on the high prevalence of founder mutations in the population, which makes it cost-effective. It also eliminates the need for provider referrals, thus, increases access to genetic services for Ashkenazi Jewish women. Eligibility for genetic services is not based on family history of HBOC-associated cancers, information that is not always available or not always accurate within family networks. The program alleviates index cases from contacting at-risk relatives and disseminating genetic information, thus, it eliminates the need for cascade screening.

However, population-based screening has also shortcomings. Individuals can have genetic testing without pre-testing counseling, which significantly compromises decisional autonomy since there is no solid informative basis for making an informed choice. When genetic testing reveals a pathogenic variant, the carrier receives post-testing counselling regarding interpretation of results, risk management options, and implications for relatives. However, when genetic testing does not identify a pathogenic variant, there is no post-testing counseling and no interpretation of the negative result as indicating an “uninformative negative” versus a “true negative” case. This may create misconceptions regarding management of cancer risk and implications for relatives, which can compromise the utility of genetic information. The program also raises concerns regarding health disparities, since Ashkenazi Jewish men, non-Ashkenazi Jews, and minority communities, e.g., Arab subgroups are not included (Michaan et al., 2021; Amar et al., 2022).

### Disseminating genetic information and implementing cascade genetic screening

The legislation in Switzerland, Korea, and Israel, and in many countries worldwide, characterizes genetic information as private and delegates the responsibility for disseminating cancer risk to the tested individual. This strategy has significant limitations in both ensuring contact with the appropriate individuals and the transmission of accurate information (Taber et al., 2015; Daly et al., 2016). Less than 50% of at-risk relatives have targeted testing and more distant relatives are often not informed due to active or passive non-disclosure (Randall et al., 2017; Committee on Gynecologic Practice, 2018; Menko et al., 2020). When pathogenic variants are identified, families may face significant barriers to cascade testing, contributing to underutilization of this service (Fehniger et al., 2013; Sharaf et al., 2013; Idos et al., 2019; Frey et al., 2020). Thus, although the law treats genetic information as belonging to the patient, promoting public health interests would require that genetic information belongs to all members of the biological family. Ownership and utility of genetic information are extremely relevant and stress ELSI dilemmas about balancing individual and family rights.

Among the 378 Swiss and Korean index cases, 6% (*n* = 17) of Swiss participants and 17% (*n* = 16) of Korean participants reported that they would rather not discuss genetic testing results with relatives. Similarly, 17% (*n* = 49) of Swiss and 19% (*n* = 17) of Korean participants who had genetic testing stated that they did not believe that genetic testing was important for their relatives. Carriers of pathogenic variants who are also affected by cancer may embrace that genetic information provides the opportunity to prevent disease in relatives, whereas healthy mutation carriers may not realize the value of this information for preventing disease in relatives (Amar et al., 2022). Narrative data from the Swiss CASCADE provide insights into the complex process of patient-mediated dissemination of testing results (Pedrazzani et al., 2022). Decisions to disseminate genetic information are governed by several logics of action, where logic is defined as the reason why and how individuals act in a given situation. Findings showed that responsibility, the feeling of having a moral duty to inform relatives, conflicted with self-preservation according to which patients inform relatives only when they feel ready and willing to disclose information about themselves. Protection of others, namely deciding whether and when it is appropriate to disclose genetic information in order not to cause harm, conflicted with respect of autonomy, when index cases wanted to respect what they thought was the relative’s will. Finally, family harmonization dictates that the decision to disseminate genetic information considers existing relationships among relatives, so as not to generate injustice or interpersonal tensions. Being exposed simultaneously to contradictory logics may restrain carriers from acting or acting without having full control over their decision. Patient-mediated dissemination of genetic information brings about ethical tensions between protecting individual rights to privacy and autonomy versus promoting public health interests, preventing disease, and downstaging cancer diagnoses.

An alternative to disseminating genetic information within the clinic-based model is that healthcare providers contact directly relatives and advocate for cascade screening, i.e., provider-mediated approach. An analysis of the Health Information Protection and Privacy Act (HIPPA) law in the U.S. (The US Department of Health and Human Services, 1996) suggested four possible scenarios under which direct contact between providers and at-risk relatives may or may not be allowed (Henrikson et al., 2020; Henrikson et al., 2021). Under these scenarios, providers can contact relatives directly with the explicit authorization of the index case, or they can contact the providers of relatives, even without such prior authorization. In the absence of authorization or an explicit objection to contacting relatives, the healthcare provider is bound by law and under no circumstances has the right to contact relatives.

In Switzerland, Korea, and Israel, the legal basis for the protection of genetic information is similar to the GINA and HIPPA (The US Department of Health and Human Services, 1996; Genetic Information Nondiscrimination Act of 2008 (GINA), 2008), leaving healthcare providers with the ethical dilemma on how to balance tensions between index cases’ autonomy and privacy versus relatives’ right to potentially life-saving information. Although the law foresees possibilities for provider-mediated dissemination of testing results, these possibilities are never or rarely enacted. Healthcare professionals in Switzerland and Israel provide a letter that can be shared with relatives. The Swiss and Israeli laws allow direct contact with relatives but with written authorization from index cases. In Switzerland, contact with relatives or their providers can be initiated without prior authorization, but after approval from a relevant Ethics Committee. In Israel, providers can only contact relatives’ providers. In Korea, providers can contact relatives’ providers but only for the diagnosis and/or treatment of the same disease.

**FIGURE 5:**
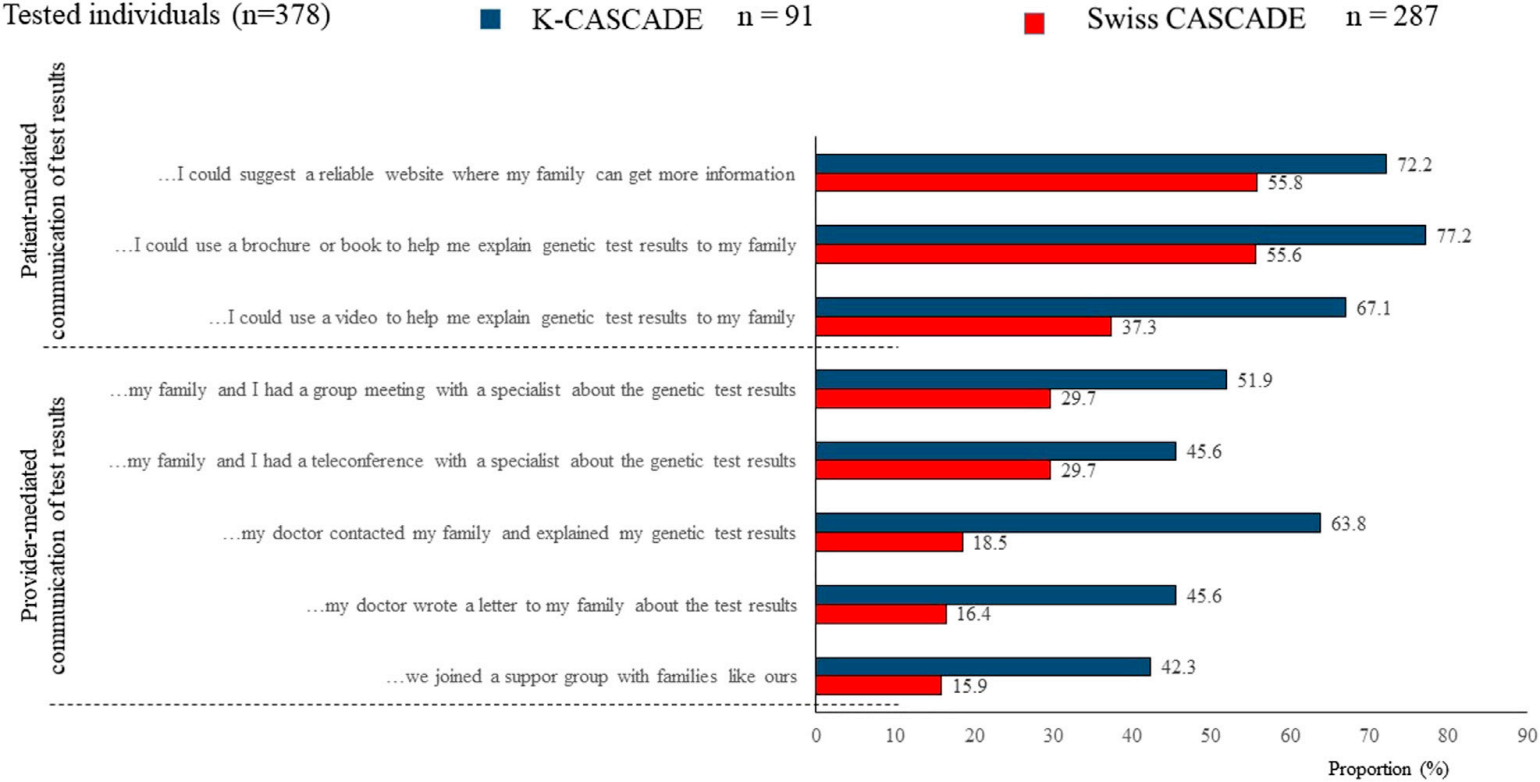
Preferences for communicating genetic testing results to relatives for Swiss and Korean participants.

Provider-mediated dissemination of genetic information should not be equated to bypassing index cases in the dissemination process. Data from *n* = 287 Swiss CASCADE index cases (had genetic testing) show that they prefer to maintain an active role in disseminating genetic information to relatives (Sarki et al., 2022b). Similarly, data from previous studies among 282 Israeli women who requested HBOC-related genetic evaluation showed that they embraced the responsibility to inform relatives while they objected to healthcare providers contacting relatives without their consent (Gilbar and Barnoy, 2012; Gilbar et al., 2016). Data from the *n* = 91 Korean K-CASCADE index cases also indicated a preference for patient-mediated communication of test results. However, about 64% (*n* = 58) of Korean index cases reported that they would like their physician to contact their family members and explain their genetic testing results (Figure 5). It is not clear if provider-mediated communication of testing results was observed due to the smaller sample of Korean participants.

## Discussion

Improving access to genetic services and cascade genetic screening are significant milestones for increasing the number of individuals who benefit from advances in genomic technologies at a global level. This paper discusses two models of accessing genetic services, a clinic-based model versus population-based screening, and two models of disseminating genetic information to relatives, the patient-mediated dissemination versus provider-mediated dissemination. Each model has its own specificities, advantages, and limitations (Table 1).

**TABLE 1:** Access and utility of genetic services.

	Clinic-based model		Population-based model
Dissemination of genetic information	Patient-mediated dissemination of testing results	Provider-mediated dissemination of testing results	Public health, media, healthcare providers
Functioning	Index case discloses testing results to relatives Clinicians’ legal duty for confidentiality	Direct contact between clinician and relatives and between clinicians	Targets populations with specific characteristics, e.g., Ashkenazi Jewish women >25 y.o. without a family history of HBOC
Advantages	Reconciles the principles of privacy, autonomy, and solidarity	Eliminates burden of index cases High-quality information	Increases access to services, eliminates need for cascade screening programs
Limitations	Burden on index cases, inequities in access to information	Legality, feasibility, acceptability, sustainability	Impairment of decision-making and lack of privacy
	Limited to risk management of tested individuals	Increases demand for number and coordination of services, effectiveness	Increases demand for coordination of services

The Israeli population-based screening program for HBOC values the public health interest and prioritizes access to genetic services over individual concerns about privacy. Cancer is defined as a reportable condition; therefore, population-based genetic screening is ethically justified (Henrikson et al., 2020). Population-based screening alleviates carriers from the responsibility to disseminate test results to relatives and eliminates the need for cascade screening programs. However, the program generates concerns regarding autonomy in decision-making due to lack of a solid information basis prior to testing and interpretation of negative results for cancer risk management. For a population-based genetic screening program to be cost-effective there should be direct links with other healthcare services that optimize the utility of genetic information by guiding the planning of adequate quantity and quality of services (National Academies of Sciences E and, 2018).

Provider-mediated dissemination of genetic information may help reconcile the inherent tension between carriers’ and relatives’ interests regarding ownership of genetic information (Wright Clayton et al., 2019). Advantages are transferring the risk communication responsibility to healthcare professionals, and providing accurate information and equitable access to care for relatives. However, this model also places a significant burden on providers, especially when the index case is not willing to cooperate. Allowing providers to directly contact relatives without prior authorization may discourage uptake of genetic testing and undermine the balance of the system, primarily the patient. A tailored approach could facilitate provider-mediated dissemination of genetic information and system–led direct contact of relatives (Roberts et al., 2018; Menko et al., 2019; Schwiter et al., 2020; Henrikson et al., 2021). This approach is also supported in our findings, where carriers prefer to actively lead the process of family communication (Gilbar et al., 2016; Griffin et al., 2019; Sarki et al., 2022a). Lack of genetic specialists (Hoskovec et al., 2018; McCuaig et al., 2018) and the need to develop and test alternative service delivery models (Buchanan et al., 2016; Randall et al., 2017) should be addressed before scaling-up this approach in cascade screening programs.

The most common practice worldwide is the clinic-based model with patient-mediated dissemination of genetic information (Israel-genetic Information Law, 2001; Rothstein, 2018; Henrikson et al., 2020; Menko et al., 2020; Whitaker et al., 2021). The responsibility of disseminating genetic information lays with index cases and may create inequity for relatives, precluding access to services when index cases do not follow through. The utility of genetic information and public interests are compromised, especially in countries with publicly-funded healthcare systems, since genetic information serves primarily the tested individual. Because of its diffusion, this model deserves particular consideration of ways to improve it. We suggest that the focus should be on how to empower index cases in the dissemination process rather than replacing them. The concepts of relational autonomy and of reflexivity can guide this process. Individuals are socially embedded and their choices are formed within the context of their social determinants and relationships (Mackenzie and Stoljar, 2000; Weller et al., 2022). Since individuals do not live in a social vacuum autonomous decision-making cannot be exercised in total independence (Stoljar, 2013). Reflexivity about one’s social practices is a crucial aspect of promoting relational autonomy in genomic decision-making. That is, supporting individuals’ capacity to recognize the complexities behind the decision of communicating (or not) testing results to relatives; reflect on their actions and those of others; and make choices according to their values and preferences in the context of their intimate family and social life. Such accounts of autonomy promote decision-making guided by an ethic of care and moral responsibility, whereby the person is respected as an individual and is also encouraged to consider her social situation and take responsibility to promote her own thriving and the thriving of others (Dove et al., 2017). The focus of healthcare systems should be on holistic person- and family-centered care, built on sound assessment, effective communication and therapeutic education, and promoting patient advocacy, self-reflection, and self-management.

## Conclusion

There is a need to address ELSI regarding genomic healthcare in light of disparities in accessing services and implementing cascade genetic screening. The CASCADE cohort highlights practices that may compromise access to services for segments of the population. The clinic-based model with patient-mediated dissemination of genetic information, maximizes privacy but compromises the utility of genetic information, especially for countries with publicly-funded healthcare systems. Population-based genetic screening programs compromises informed decision-making and the ability for self-reflection and self-determination. Within these models, we suggested enhancing relational autonomy and individual reflexivity as key ingredients of promoting the utility of genetic information, while complying with privacy laws, ethical boundaries, and healthcare systems.

## Members of the Swiss CASCADE Consortium

Monica Aceti, Vasiliki Baroutsou, Chang Ming, Carla Pedrazzani, Islam Salikhanov, Mahesh Sarki, Reka Schweighoffer (Department of Clinical Research, University of Basel); Souria Aissaoui, Ashley Machen (Breast Center, Cantonal Hospital Fribourg, Fribourg; GENESUPPORT, The Breast Centre, Hirslanden Clinique de Grangettes, Geneva); Murat Aykut, Ursina Zürrer-Härdi (Department of Medical Oncology, Cantonal Hospital Winterthur, Winterthur); Fulvia Brugnoletti (Unit of Oncogenetics, Division of Oncology, University Hospitals of Geneva); Nicole Bürki, Viola Heinzelmann-Schwarz, Eveline Schönau (Women’s Clinic, University Hospital Basel, Basel); Pierre O. Chappuis (Unit of Oncogenetics, Division of Oncology, University Hospitals of Geneva; Division of Genetic Medicine, University Hospitals of Geneva, Geneva); Muriel Fluri, Manuela Rabaglio, Saskia Schlootz (Department of Medical Oncology, Inselspital, Bern University Hospital, Bern); Rossella Graffeo (EOC, Oncology Institute of Southern Switzerland (IOSI), Bellinzona); Karl Heinimann (Institute of Medical Genetics and Pathology, University Hospital Basel, Basel; Research Group Human Genomics, Department of Biomedicine, University of Basel, Basel); Andrea Kaiser-Grolimund (Institute of Social Anthropology, University of Basel); Helen Koechlin (Institute of Clinical Psychology, University of Zurich); Christian Kurzeder (The Breast Center, University Hospital Basel); Christian Monnerat (Department of Medical Oncology, Hospital of Jura, Delémont); Nicole Probst-Hensch (Swiss Tropical and Public Health Institute); Simon Wieser (ZHAW School of Management and Law, Winterthur Institute of Health Economics).

## Members of the K-CASCADE Consortium

Joon Jeong (Department of Surgery, Gangnam Severance Hospital, College of Medicine, Yonsei University); Mi Sook Jung (College of Nursing, Chungnam National University); Jisun Kim (Department of Surgery, Asan Medical Center, College of Medicine, University of Ulsan); Sung-Won Kim (Dairim St. Mary’s Hospital); Myong Cheol Lim (Division of Tumor Immunology, Center for Gynecologic Cancer, Research Institute and Hospital, National Cancer Center); Eun-Ji Nam (Deptarment of Obstetrics and Gynecology, College of Medicine, Yonsei University); Hyung Seok Park (Department of Surgery, Severance Hospital, College of Medicine, Yonsei University); JiSoo Park (Hereditary Cancer Clinic, Cancer Prevention Center, Yonsei Cancer Center, College of Medicine, Yonsei University); Sanghyun Park (Department of Computer Science, Yonsei University); Jai Min Ryu (Division of Breast Surgery, Department of Surgery, Samsung Medical Center, School of Medicine, Sungkyunkwan University).
